# Pulmonary adenofibroma: MSCT and clinicopathological analysis of three case reports and a literature review

**DOI:** 10.1097/MD.0000000000050403

**Published:** 2026-08-21

**Authors:** Wang Chen, Kang Liu, Yang Zhao, Chen-chen Liu, Meng-yu Cao, Xiao-juan Wang

**Affiliations:** a Imaging Center, The First Affiliated Hospital, College of Clinical Medicine of Henan University of Science and Technology, Luoyang, China; b Pathology Department, The First Affiliated Hospital, College of Clinical Medicine of Henan University of Science and Technology, Luoyang, China.

**Keywords:** imaging, pathology, pulmonary adenofibroma

## Abstract

**Rationale:**

Pulmonary adenofibroma (PAF) is a rare, benign lung tumor, classified as a primary fibroepithelial tumor of the lung.

**Patient concerns:**

This study reports 3 cases of pulmonary adenofibroma (PAF). Case 1 was a solitary tumor presenting with a cough; Case 2 was a solitary tumor manifesting as chest tightness and pain; Case 3 was a multiple-lesion variant in an asymptomatic patient.

**Diagnoses:**

Pulmonary lesions were detected in all patients by computed tomography (CT) and were ultimately diagnosed as PAF through histopathological analysis.

**Interventions:**

Cases 1 and 2 underwent video-assisted thoracoscopic wedge resection, while Case 3 received conservative management with active surveillance.

**Outcomes:**

Cases 1 and 2 recovered well postoperatively, with no signs of recurrence or metastasis during follow-up. Case 3 remained stable, with some nodules demonstrating slow growth and no evidence of metastasis.

**Lessons:**

Pulmonary adenofibroma is readily confused with hamartoma and solitary fibrous tumor, and definitive diagnosis relies on histopathological examination.The tumor demonstrates indolent biological behavior and a favorable prognosis; therefore, overtreatment should be avoided in clinical practice.

## 1. Introduction

Pulmonary adenofibroma (PAF) is a mixed epithelial-mesenchymal tumor originating from lung tissue with an unclear origin. Its microscopic morphology resembles that of adenofibromas of the female genital tract and fibroadenomas of the breast. The tumor was first named by Suster and Moran in 1993.^[1]^ Clinically, pulmonary adenofibroma is extremely rare, with fewer than 50 cases reported worldwide to date.^[2]^ It has not yet been included in the WHO classification of lung tumors.^[3]^ The etiology of PAF remains unknown, and it is generally considered unrelated to smoking. Current clinical understanding is insufficient, leading to potential misdiagnosis or confusion with other tumors. This study retrospectively analyzes the clinical data of 3 PAF patients from our institution and reviews the literature to summarize the imaging and clinicopathological features of this tumor, aiming to assist clinicians in further improving their recognition of PAF.

## 2. Case presentation

### 2.1. Case 1

**Figure 1.**
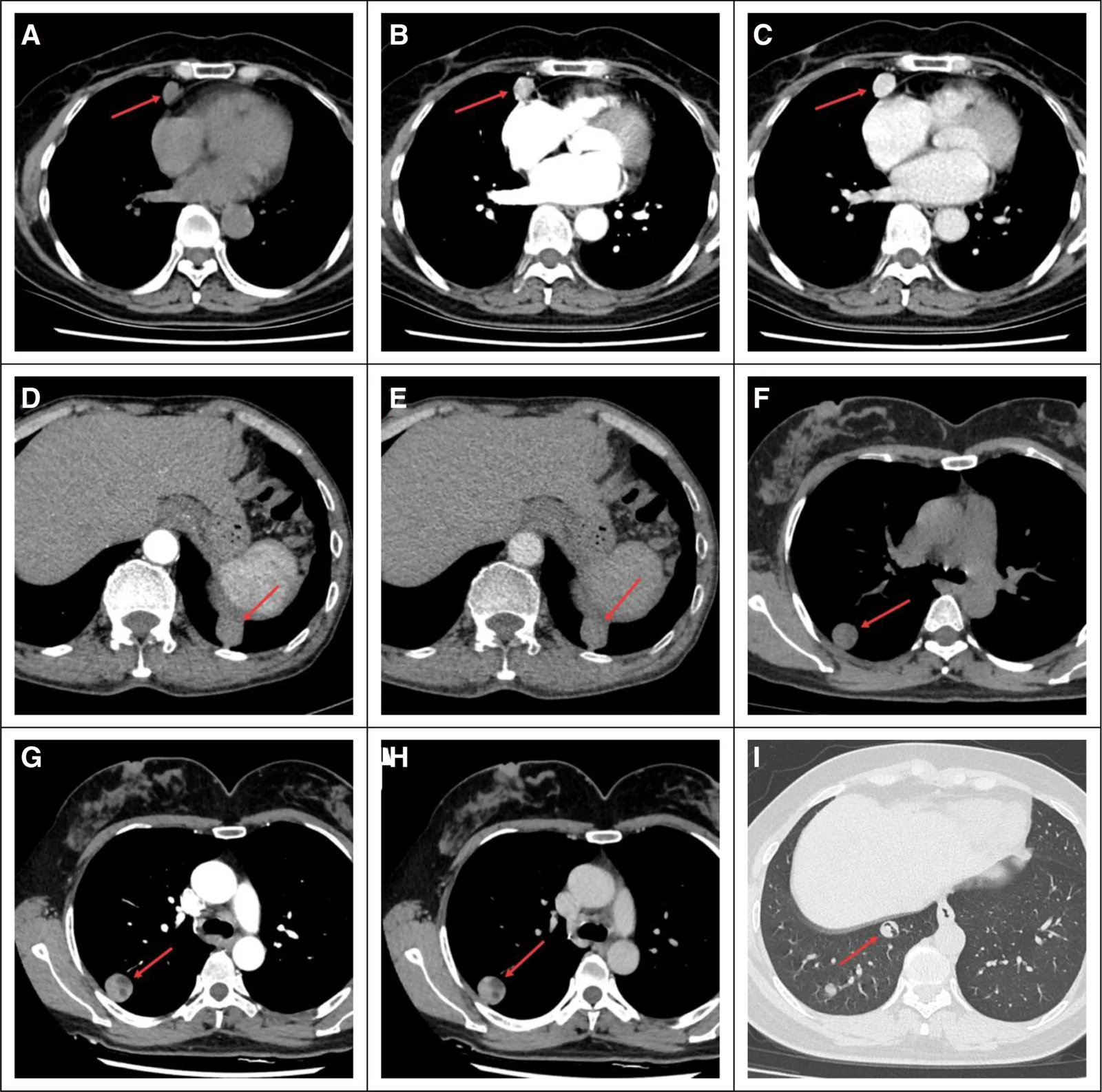
Chest CT images of pulmonary adenofibroma. Case 1 (A,B,C) A 60-year-old female. Images demonstrate chest CT mediastinal window, contrast-enhanced arterial phase, and venous phase, respectively. A well-defined solid nodule approximately 18 mm in diameter is seen in the medial segment of the right middle lobe, showing prominent but heterogeneous enhancement with more pronounced enhancement during the venous phase. Case 2 (D, E) A 66-year-old male. Images show contrast-enhanced chest CT mediastinal window during arterial and venous phases. A solid nodular shadow measuring about 26 × 20 mm is observed in the posterior basal segment of the left lower lobe. The margins are relatively smooth but poorly demarcated from the left diaphragm. Case 3 (F–H) A 44-year-old female. Images display chest CT mediastinal window, contrast-enhanced arterial phase, and venous phase, respectively. A heterogeneous round-like lesion approximately 22 mm in diameter is noted in the posterior segment of the right upper lobe. Patchy hypodense areas with no significant enhancement are visible on contrast-enhanced scans. (I) A round lesion in the apical segment of the right lower lobe, measuring about 14 mm in diameter, demonstrates internal gas-density shadows.

A 60-year-old female was admitted with a one-month history of cough and a pulmonary mass discovered on physical examination 2 days prior. She was previously healthy with no history of underlying diseases such as hypertension or diabetes, and no history of smoking or alcohol use. All laboratory parameters were within normal limits. Computed tomography (CT) revealed a high-density nodule, approximately 18 × 14 mm in diameter, in the medial segment of the right middle lobe. The nodule had clear borders and showed significant but heterogeneous enhancement, with pre-contrast CT values around 34 HU, 100 HU in the arterial phase, and 133 HU in the venous phase. The CT diagnosis suggested sclerosing pneumocytoma (Fig. 1A–C). After a multidisciplinary evaluation,video-assisted thoracoscopic (VATS) right middle lobectomy was performed. The resected specimen was sent for pathological examination, which confirmed the diagnosis of PAF. The patient received no additional treatment, had an uneventful postoperative recovery, and showed no evidence of recurrence during 1 year of follow-up.

### 2.2. Case 2

A 66-year-old male was admitted with a half-month history of intermittent chest tightness. He had a history of coronary heart disease. A CT scan from an external hospital revealed a mass in the left lower lobe, suspected to be malignant. No abnormalities were detected in the laboratory tests.Contrast-enhanced chest CT showed a solid nodular shadow in the posterior basal segment of the left lower lobe, measuring approximately 26 mm × 20 mm × 14 mm, with relatively smooth margins, adjacent to the left diaphragm. It showed mild enhancement on contrast-enhanced CT, with CT values of approximately 58 HU and 53 HU in the arterial and venous phases, respectively. Imaging findings suggested a neoplastic lesion (Fig. 1D,E). Following a multidisciplinary team evaluation, the patient underwent a video-assisted thoracoscopic surgery wedge resection of the left lower lobe. Histopathological examination of the resected tissue confirmed the definitive diagnosis. The patient had an uneventful recovery with no signs of recurrence during a six-month follow-up period.

### 2.3. Case 3

A 44-year-old female had no respiratory-related symptoms or complaints. She was previously healthy with no smoking history. One year prior, a chest CT during a physical examination at our hospital revealed multiple nodules in both lungs, ranging in diameter from 6 to 23mm. The larger nodule was located in the posterior segment of the right upper lobe, measuring approximately 23 mm in diameter. On contrast-enhanced CT, the pulmonary nodules showed mild to moderate heterogeneous enhancement, with patchy non-enhancing low-density areas within some nodules (Fig. 1F–H). Some nodules contained gas-attenuation areas (Fig. 1I). Subsequent PET-CT demonstrated multiple bilateral pulmonary nodules with mildly increased FDG uptake (SUVmax ≈ 1.6).Tumor markers were within normal limits. No other specific procedures or treatments were performed. For further evaluation, a CT-guided percutaneous needle biopsy of the lesion in the posterior segment of the right upper lobe was performed. Pathological examination of the biopsy specimen confirmed the diagnosis of pulmonary adenofibroma. During the one-year follow-up, slow growth was observed in a subset of the nodules. For instance, the lesion in the posterior basal segment of the left lower lobe demonstrated a slight increase in size, from an initial diameter of 15 mm to approximately 17 mm on the most recent CT scan.

## 3. Pathological findings

Gross examination: Cases 1 and 2 were single nodules, 1.4–3 cm in diameter. Macroscopically, the nodules were well-circumscribed, with a gray-white cut surface, uniform texture, and medium firmness. Case 3 (right upper lobe) consisted of biopsy cores, 0.1–0.5 cm long and 0.1 cm in diameter.

**Figure 2.**
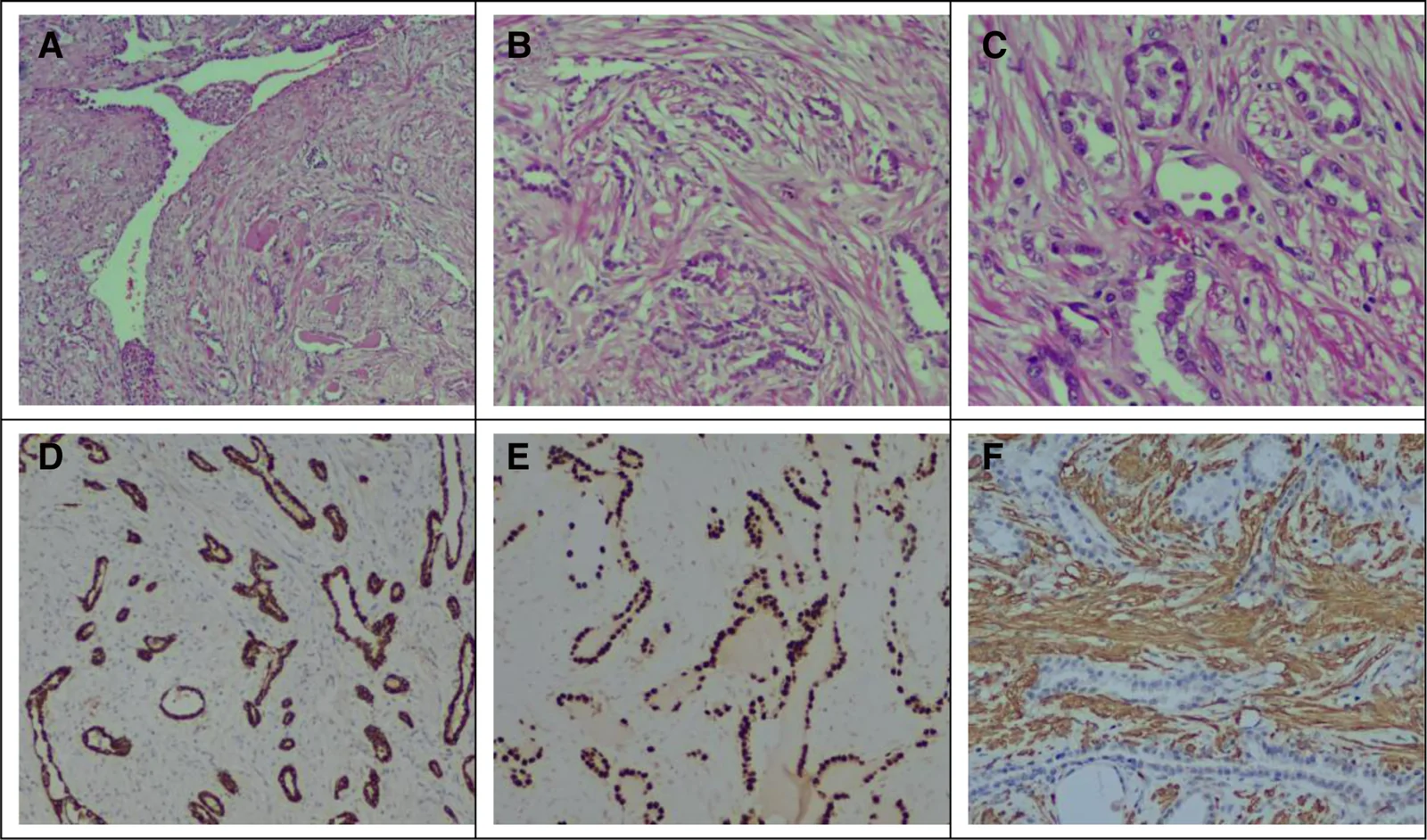
Histopathological results. (A)The tumor is composed of a mixture of epithelial and mesenchymal components (H&E,×40), (B) Tumor stroma composed of sheets of interlacing spindle cells with collagenization; gland-like spaces are interspersed (H&E,×100), (C) Mesenchymal component of tumor consisting of spindle/plump spindle cells with fibroblastic/myofibroblastic characteristics (H&E,×200), (D) Epithelial components express CK (IHC,×100), (E) Epithelial components show positive TTF-1 expression (IHC,×100), (F) Stromal components show positive SMA expression (IHC, ×100).

Microscopic examination: The tumors were composed of a mixture of epithelial and mesenchymal components with clear boundaries, showing lobulated, slit-like, papillary, and cystadenomatous structures (Fig. 2A). The epithelial tumor cells were arranged in glandular tubules of varying sizes, with anastomosing glands interspersed among spindle-shaped stromal cells. Some glandular lumens contained colloid-like, eosinophilic, structureless material. The glands were lined by a single layer of short, ciliated, low columnar or cuboidal bronchial or alveolar-type epithelium, with eosinophilic or clear cytoplasm (Fig. 2B). The stromal cells were elongated or plump spindle-shaped, tending to surround the glands, with areas of collagenization (Fig. 2C). Both epithelial and stromal cells showed no significant atypia or mitotic activity.

Immunohistochemistry (IHC): The epithelial components expressed cytokeratin (CK) (Fig. 2D) and TTF-1 (Fig. 2E). The epithelial component in Case 1 was negative for Napsin A, while the components in Cases 2 and 3 were positive for Napsin A. The immunohistochemical phenotype of the stromal components varied among cases: the stroma in Case 1 expressed SMA (Fig. 2F); the stroma in Case 2 was immunoreactive for STAT6 and Bcl-2, with focal positivity for CD34 and PR; the stroma in Case 3 expressed Vimentin, ER, and SMA. The Ki-67 proliferation index was low in all cases, approximately 1–2%. Based on these immunohistochemical features combined with the histological morphology, all cases were diagnosed as pulmonary adenofibroma.

## 4. Discussion

A search of the PubMed database using the keywords “Pulmonary & adenofibroma” and “lung & adenofibroma,” and searches of the China National Knowledge Infrastructure (CNKI), Wanfang Database, and Chinese Biomedical Literature Database (CBM) (from inception to August 2025) using the keyword “pulmonary adenofibroma” yielded 34 relevant articles (22 English articles and 12 Chinese articles). Consolidating the literature, PAF often lacks typical clinical symptoms, with most cases discovered incidentally during physical examination. Some patients present with pulmonary symptoms such as cough or chest pain. Most reported PAF cases have follow-up data, and none have reported recurrence or tumor-related death.^[4]^

The exact origin of PAF remains debated. Its tissue origin is still unclear, and whether PAF can be considered a distinct tumor type is debated. Some viewpoints suggest it should be classified as a subtype of hamartoma or solitary fibrous tumor (SFT).^[5]^ Some studies propose that the pathological nature of PAF may be related to the hamartoma spectrum. Suster and Moran^[1]^ suggested that the stromal component of these tumors is arrested at an immature fibroblastic stage and does not differentiate into the cartilage or smooth muscle commonly seen in classic hamartomas, supporting the classification of PAF as a special type of hamartomatous lesion. Other studies argue that PAF is a neoplastic rather than hamartomatous lesion.Fusco et al^[6]^found recurrent NAB2-STAT6 gene fusions in the stroma of PAF, a hallmark of SFT, with 71% of cases showing estrogen receptor-α overexpression. In their series, all STAT6-positive PAFs by immunohistochemistry carried the fusion gene, while STAT6-negative cases did not, confirming STAT6 immunohistochemistry as a reliable surrogate marker for the fusion. Based on these findings, Fusco et al proposed that PAFs are neoplastic lesions harboring the molecular hallmark of SFT and may represent a histological variant of intrapulmonary SFT. Our Case 2 showed nuclear STAT6 expression and a paucicellular collagen-rich stroma, which is consistent with this profile.Due to the rarity of cases, its relationship with SFT requires further study.

The detailed data for the 3 present cases and the relevant cases from the literature are summarized in Table 1.The 3 patients included in this study (one male, 2 females), aged between 44 and 66 years, align with the reported epidemiological characteristic of PAF occurring predominantly in middle-aged and elderly populations (40–70 years).^[6,11]^ Regarding tumor size and distribution, the solitary nodules in Cases 1 and 2 measured 18mm and 26mm respectively, and the multiple nodules in Case 3 ranged from 6 to 23mm in diameter, consistent with the reported tumor diameter range of 5–45mm. This study did not include younger patients, while existing literature suggests that although rare, younger patients can present with larger tumors (diameter > 45mm).^[5,11]^ The distribution of lesions in our cases showed one each: solitary in the right middle lobe, solitary in the left lower lobe, and multiple in both lungs. This is largely consistent with the literature description of “mostly solitary lesions (left lung more than right, lower lobe more than upper lobe), rarely multiple lesions.”^[7,8]^ No imaging features suggestive of malignancy were observed in these 3 cases, a finding that is consistent with the typically benign biological behavior of PAF as documented in the literature.^[9]^

**Table 1:** Summary of clinical characteristics of PAF from the literature.

Author	Year	Case	Gender	Age	Size(cm)	Location	Strengthen features	Epithelial component (pos)	Stromal component (pos)	Stromal component (neg)
Kumar^[5]^	2014	3	M	25	4.5	left lower		CK, EMA, TTF-1	Vimentin, CD34	S-100, Bcl-2
			F	40	5	left lower		CK7, TTF1, NapsinA	SMA, Vimentin	S-100, CD34, Bcl-2, HMB45
			F	55	2.2	left upper		CK7, TTF1, NapsinA	SMA, Vimentin	S-100, CD34, Bcl-2, HMB45
Hao^[7]^	2016	1	F	57	0.2–1.5	Multiple bilateral lung nodules; largest in left upper lobe		CK7, TTF-1 , NapsinA,	vimentin, desmin, SMA, h-CALD	CD34, CD 117, CD99
Kim^[8]^	2020	1	F	71	1.7	left lower	No enhancement	CK7, STAT6	SMA, ER, CD34, Bcl-2, STAT6	
Wang^[9]^	2010	1	M	55	1.2	left lower	No enhancement	Cam5.2, EMA, TTF1	Vimentin, CD34	
Liang^[2]^	2021	7	F	54	2.3	left lower		CK, TTF1, EMA		CD34, S-100, SMA, desmin
			M	62	2.5	right upper		CK, TTF1, EMA		ER, PR, CD34, STAT6, S-100, SMA, desmin
			F	46	0.5	right upper		CK, TTF1	ER, PR, CD34, SMA, desmin	STAT6
			F	47	1.2	left upper		TTF1, EMA	ER, Bcl-2	PR, CD34, STAT6, SMA, desmin
			F	66	1	left lower		CK, EMA	CD34	ER, PR, STAT6, SMA, desmin
			F	45	2.7	left lower		CK, TTF1, EMA	ER, PR, Bcl-2, CD34, SMA, desmin	STAT6, S-100
			M	34	2	right upper		CK	ER, PR, SMA, desmin	CD34, STAT6, S-100
Shen^[10]^	2022	1	M	70	6.5	left lower	Mild enhancement	CK, EMA	Bcl-2, CD34, STAT6, Vimentin	
Our date		3	F	60	1.8	right middle	Marked enhancement	CK, TTF-1, NapsinA,	SMA, ER	
			M	66	2.6	left lower	Mild enhancement	CK, TTF-1, Napsin A	STAT6, Bcl-2, CD34, PR	SMA, desmin, S-100
			F	44	0.6–2.3	2 right upper 1 right middle 6 right upper 4 left lower	4 Mild enhancement 8 Moderate enhancement 1 No enhancement	CK, TTF-1, Napsin A	Vim, ER, SMA	

Radiologically, PAF typically presents as a well-defined, solitary pulmonary nodule, often located within the lung parenchyma near the pleura, exhibiting features of a benign tumor on imaging. In our 3 cases, the enhancement patterns of the pulmonary nodules on contrast-enhanced CT were diverse, which is generally consistent with previous literature reports.^[10]^ This variation in enhancement may be closely related to the histological composition of PAF. PAF is composed of both stromal and epithelial components. Sheets of interlacing, proliferative stroma often compress glandular lumens and form slit-like structures, making these areas relatively dense and vascularized, thus appearing as pronounced or moderate enhancement on CT (e.g., Case 1 nodule reached 100 HU in the arterial phase). Conversely, areas predominantly consisting of abundant glandular structures filled with colloid-like, eosinophilic, structureless material and sparse stroma present as low-density areas with no significant enhancement on CT. The spatial distribution differences of these components within the tumor constitute the pathological basis for its heterogeneous imaging appearance, which is significant for CT diagnosis and differential diagnosis of this disease. Furthermore, the presence of internal gas attenuation observed in some nodules of Case 3, a feature rarely reported in previous literature on PAF, may be attributed to communication with peripheral airways or secretory changes. This finding merits consideration as a novel addition to the spectrum of imaging features of PAF.

Pulmonary adenofibroma is a mixed epithelial-mesenchymal tumor composed of abundant glandular and stromal components. Glandular lumens may be compressed into slit-like structures, often containing eosinophilic secretions; they are lined by a single layer of cuboidal or ciliated columnar epithelium, possessing bronchiolar/alveolar epithelial attributes. The stroma is composed of fibroblasts/myofibroblasts with uneven density distribution, and collagen components are often observed in the background.^[12]^ Prominent collagenous areas were observed in the tumor components of all cases in this series. Many cases also show lobulated and papillary structures, similar to fibroadenomas of the breast and adenofibromas of the ovary.^[2]^ The IHC characteristics of PAF are highly reproducible. The epithelial components consistently express CK, EMA, and TTF-1, indicating pulmonary origin. In contrast, the stromal components show significant variation, with possible expression of CD99, CD34, BCL-2, SMA, ER, and STAT6 to varying degrees. The Ki-67 proliferation index is consistently low (<2%), reflecting the indolent biological behavior of this tumor.The microscopic histomorphology and IHC phenotypes of the 3 cases in this study are largely consistent with literature reports, supporting its classification as an indolent, biphasic pulmonary tumor.

PAF primarily needs to be differentiated from hamartoma and solitary fibrous tumor (SFT). Hamartomas can also present as well-defined, smooth-edged nodules. However, microscopic examination reveals histological components not only including fibrous stroma and epithelium but also often cartilage, smooth muscle, fat, and other tissues. In contrast, PAF has a relatively simple composition, consisting only of glandular epithelium and fibrous stroma. Radiologically, due to their diverse components, hamartomas may show fat attenuation or calcification on CT, while PAF mostly presents as a homogeneous density nodule. Some PAFs may appear heterogeneous due to internal structural differences but typically lack the calcifications common in hamartomas. PAF also needs differentiation from SFT: both PAF and SFT show spindle-shaped stromal components microscopically. However, unlike SFT, the stromal cells in PAF often express ER and PR, which are not expressed in SFT. Radiologically, SFT is often a pleural-based, markedly enhancing solitary mass, while PAF is mostly located within the lung parenchyma with varied enhancement patterns. In summary, although imaging can provide clues regarding nodule morphology, density, and enhancement patterns, its manifestations often lack specificity and have limitations in the differential diagnosis of PAF. Definitive diagnosis still relies on pathological and immunohistochemical examination.

In conclusion, PAF is a rare benign lung tumor with a favorable prognosis; no cases of recurrence or metastasis have been reported in previous studies. For solitary or multiple nodules found on lung CT, especially when heterogeneous enhancement signs such as patchy non-enhancing low-density areas are present within the nodule, combined with relevant clinical manifestations, PAF should be considered as a possible diagnosis. However, definitive diagnosis still depends on pathological and immunohistochemical examination.

The present study has certain limitations, including the small sample size and the relatively short follow-up period (6–12 months). Due to the limited follow-up duration, only short-term outcomes can be confirmed to be consistent with the benign biological behavior of PAF, while the long-term prognosis remains to be further elucidated, particularly in conservatively managed multifocal cases. In multifocal Case 3, only the largest nodule was pathologically confirmed; although the remaining nodules demonstrated imaging features and follow-up findings highly consistent with the confirmed lesion, they lacked pathological verification, and the possibility of histological heterogeneity cannot be completely excluded. The biopsy specimen contained both epithelial and stromal components with an immunohistochemical profile consistent with PAF, supporting the diagnosis. Future efforts should expand surveillance and collect more cases to further investigate this tumor and to help clinicians gain deeper insights into this disease.

## Author contributions

**Conceptualization:** Wang Chen.

**Writing – original draft:** Wang Chen, Kang Liu.

**Writing – review & editing:** Wang Chen, Kang Liu.

**Methodology:** Kang Liu, Yang Zhao, Chen-chen Liu, Meng-yu Cao, Xiao-juan Wang.
